# Prevalence of Tobacco Smoking and Its Association With Disease Severity Among Patients With Psoriasis in China: A Cross-Sectional Study

**DOI:** 10.3389/fmed.2022.883458

**Published:** 2022-05-12

**Authors:** Lei Wei, Siting Chen, Zhan Zhang, Le Kuai, Rui Zhang, Ning Yu, Yuling Shi, Bin Li, Ruiping Wang

**Affiliations:** ^1^Clinical Research Center, Shanghai Skin Diseases Hospital, Tongji University, Shanghai, China; ^2^Department of Dermatology, Yueyang Hospital of Integrated Traditional Chinese and Western Medicine, Shanghai University of Traditional Chinese Medicine, Shanghai, China; ^3^National Clinical Research Center for Skin and Immunity Diseases, Beijing, China; ^4^Department of Dermatology, Shanghai Skin Diseases Hospital, Tongji University, Shanghai, China

**Keywords:** tobacco smoking, psoriasis severity, current smoker, former smoker, smoking intensity, smoking duration

## Abstract

**Introduction:**

Tobacco smoking is associated with the onset and severity of psoriasis, and understanding the prevalence of tobacco smoking among patients with psoriasis is critical due to its high physical and mental influence and heavy disease burden. However, evidence on the association between tobacco smoking and psoriasis severity is still limited in China.

**Objectives:**

The objective of this study was to examine the prevalence of tobacco smoking and explore the association between tobacco smoking and diseases severity among patients with psoriasis.

**Methods:**

A total of 4,529 patients with psoriasis in 200 selected hospitals were recruited in China between January 2020 and September 2021. Detailed information covering demographic feature, tobacco smoking, and psoriasis history were collected through an electronic questionnaire, and clinical data were extracted from the health information system (HIS). SAS 9.4 was used for data analysis, and a *p*-value of <0.05 was considered statistically significant.

**Results:**

The prevalence of tobacco smoking was 30.8%, with 24.6% for current smoking. The average Psoriasis Area and Severity Index (PASI) score for patients with psoriasis was 9.4, with male patients having a higher PASI score than female patients. The odds ratio (OR) of former tobacco smoking prevalence was 1.5 [95% confidence interval (CI): (1.0–2.3)] for PASI score (3.0–7.0), 2.2 for PASI score (7.1–13.0), and 4.2 for PASI score >13, when compared with patients with PASI score < 3.0. Similarly, the OR of current tobacco smoking prevalence was 1.8 [95% CI: (1.5–2.2)] for PASI score (3.0–7.0), 1.9 for PASI score (7.1–13.0), and 3.1 for PASI score >13, when compared with patients with PASI score <3.0. The Spearman correlation analysis indicated that both tobacco smoking intensity and smoking duration were positively correlated with psoriasis severity (*p* < 0.05).

**Conclusion:**

The prevalence of tobacco smoking was high, especially among male patients with psoriasis and those with senior high education. Tobacco smoking was positively associated with psoriasis severity; moreover, both smoking intensity and smoking duration were positively correlated with the severity of psoriasis in a dose-dependent fashion.

## Introduction

Psoriasis is a common chronic systemic skin disease induced by a combination of environmental and genetic factors (1, 2). Globally, the prevalence of psoriasis in adults ranges from 0.51 to 11.43% (3). A recent epidemiological investigation reveals that psoriasis affects ~125 million population worldwide and the prevalence of psoriasis is increasing gradually (4). Previous studies have indicated that the Chinese population has relatively lower psoriasis prevalence, but in recent years, the prevalence of psoriasis in China has increased from 0.16% in 1982 to 0.47% in 2012. It can be speculated that there are about 6 million patients with psoriasis in the 1.4 billion Chinese population (5, 6). Clinically, psoriasis is characterized as erythematous plaques, silvery scales on the skin lesion, accompanied by joint damage and multiple organs' dysfunctions (7, 8). Psoriasis adversely affects the quality of life and work efficiency of patients, leads to severe economic burden as well (9–11), and is considered to be a serious global problem.

In recent years, a growing number of studies have indicated that environmental factors are vital triggers for the initiation of psoriasis, and tobacco smoking is one of the key environmental pathogenic factors which affects the onset of psoriasis (12, 13). Bertesi et al. reported that the onset of psoriasis is associated with the role of keratinocytes, fibroblasts, immune cells, and inflammatory factors (14), and the psoriasis severity is closely related to the inflammation. In addition, tobacco smoking can induce oxidative stress, free radical damage, increase vascular endothelial dysfunction, and immune cell activation, and can interact with the key signaling pathways in psoriasis pathogenesis. Multiple studies have been implemented previously to investigate the association between psoriasis and tobacco smoking, and tobacco smoking is proved as an independent risk factor that accounts for the increased prevalence of psoriasis (15, 16). Therefore, advocating tobacco smoking cessation is crucial to curb the elevated prevalence and the severe influence of psoriasis in the world.

Tobacco smoking is the single most avoidable cause of death and disability globally, and it is estimated that there are 1.2 billion tobacco smokers in the world. In recent years, tobacco smoking in low- and middle-income countries is increasing rapidly, and the prevalence of tobacco smoking among men in Asian countries is now far higher than in Western countries, with 45% in India, 53% in Japan, 63% in China, 69% in Indonesia, and 73% in Vietnam (17). As the largest tobacco producer and consumer in the world, China is facing a public crisis with an estimation of 320 million tobacco smokers with current smoking prevalence (18). The Global Adult Tobacco Survey (GATS) conducted in 2010 indicates that nearly 1 million smokers died from tobacco-related diseases, and about 52% of non-smokers are exposed to second-hand tobacco smoke in China (19). Due to the tobacco-induced heavy disease burden, and its association with psoriasis, it is critical to understand the prevalence of tobacco smoking among patients with psoriasis for future tobacco control in this targeted population. However, evidence on the prevalence of tobacco smoking and, especially, its association with psoriasis severity is still limited in China (20–22).

In this study, we implemented a cross-sectional investigation that covered 200 hospitals in China. We aimed to understand the prevalence of tobacco smoking among patients with psoriasis and to explore the association between tobacco smoking and psoriasis severity. Accurate assessment of potentially modifiable factors is critical for psoriasis management, and confirmation of the relationship between tobacco smoking and psoriasis severity indicates the need for behavior modification among patients with psoriasis in the future.

## Methods

### Study Population

**Figure 1:**
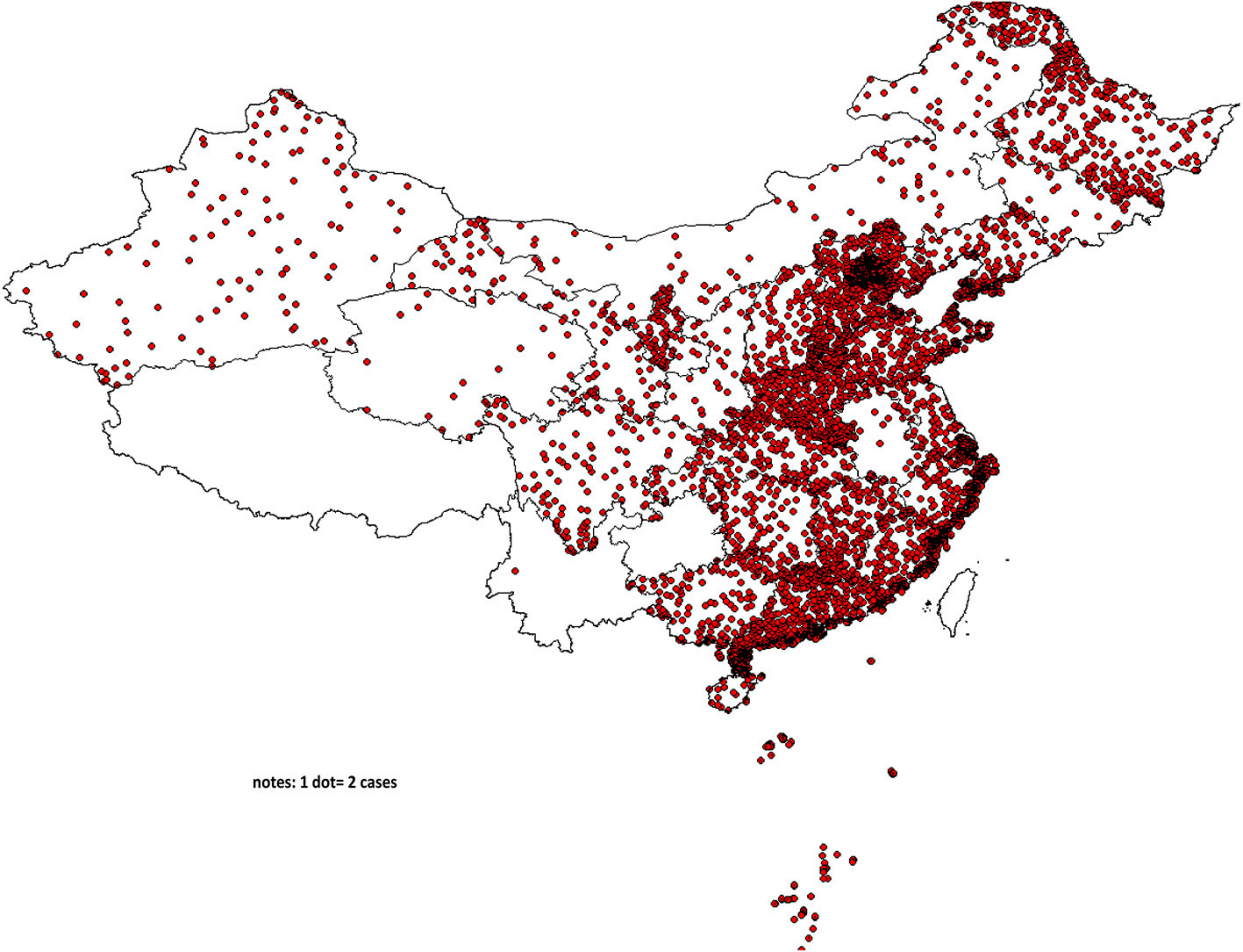
Regional distribution of information collection for 4529 patients in China.

Data in this study were originated from the National Clinical Research Center for Skin and Immune Diseases (NCRCSID) in China. The NCRCSID was established in January 2020 and was aimed to build a database in China to collect extensive real-world data on psoriasis from 200 hospitals selected in all seven regions of China (Figure 1). In this study, 200 hospitals were selected by a multistage quota sampling manner. First, 30% of cities (prefectures or districts) among the 31 provinces, autonomous regions, and municipalities in China (except Hong Kong, Taiwan, and Macao) were selected randomly. Second, almost 10% of all tertiary hospitals and secondary hospitals in the selected cities were contacted and recruited in a voluntary and convenient manner, and finally, 200 hospitals were recruited in total. In this study, all patients with psoriasis aged ≥18 years were recruited from January 2020 to September 2021, and all patients had a clinical diagnosis of psoriasis and received a standardized personal interview with informed consent. In the data collection process, a structured questionnaire was completed, and the investigators applied the Psoriasis Area and Severity Index (PASI) to measure the psoriasis severity. Eventually, personal information of each participant was encrypted and kept anonymous in the psoriasis database, and finally, 4,529 patients with psoriasis were included in the analysis.

This study was approved both by the Institutional Review Board of Peking University First Hospital, Beijing, China (2020-255), and the Review Board of Shanghai Skin Diseases Hospital, Shanghai, China (2021-27). Informed consent was signed by each participant before the questionnaire interview, and this study was strictly performed in accordance with the guideline of the STROBE statement and the Declaration of Helsinki.

### Diagnosis, Inclusion, and Exclusion Criteria of Psoriasis

In this study, patients with psoriasis were clinically diagnosed, and all patients with psoriasis met psoriasis diagnostic criteria based on the Chinese clinical dermatology, which was in line with the global guideline for psoriasis diagnosis and treatment. The skin lesions of psoriasis are manifested as red inflammatory papules, patches of varying sizes covered with multiple layers of silvery-white scales, with punctate hemorrhages visible on the removal of the scales (Auspitz's sign). In this study, the inclusion criteria of psoriasis were patients who met the clinical criteria for psoriasis and patients aged over 18 years for both genders, and patients with neurological disorders, psychiatric abnormalities, or unable to provide informed consent were excluded.

### Data Collection

Detailed information in this study was collected by an electronic questionnaire from January 2020 to September 2021. Data were collected by local dermatologists from the 200 selected hospitals after the uniform training procedures to guarantee the authenticity and validity of the data. The main contents of the questionnaire were as follows: (1) demographic characteristics, including name, gender, age, marital status, education, regions, medical insurance, and body mass index (BMI); (2) tobacco smoking status covering current/former smoker, years of tobacco smoking, number of daily consumed cigarettes, and tobacco smoking cessation; and (3) information of psoriasis onset and diagnosis, including PASI score, seasonality of psoriasis aggravation, types of psoriasis, and family history of psoriasis.

### Definition and Index Calculation

In this study, smokers were defined as those who smoked at least 100 cigarettes in their lifetime (19), and smoking cessation was defined as a smoker who has quit smoking for at least 3 months. We defined current smokers as those smokers who were still smoking at the time of investigation, and former smokers as those smokers who had abstained from smoking for over 3 months at the time of investigation.

Tobacco smoking data were available regarding the collected information on the number of cigarettes smoked, age for tobacco smoking initiation, and year of tobacco smoking. Smoking duration was calculated as age at investigation minus age of the first started regular tobacco smoking for current smokers, and as age at smoking cessation minus age of the first started regular tobacco smoking for former smokers; then, smoking duration was divided into three groups, namely, <10 years, 10–20 years, and over 20 years. The smoking intensity was defined as the number of cigarettes smoked every day and was dichotomized as <20 cigarettes/day and ≥20 cigarettes/day. Smoking cessation years were calculated as the age at investigation minus the age at smoking cessation initiation among former smokers, and were divided into <10 years and equal or over 10 years.

In this study, the BMI of patients with psoriasis was categorized as lower body weight (<18.5), normal weight (18.5–23.9), overweight (24.0–27.9), and obesity (≥28.0). The education status of patients with psoriasis was classified as junior high and lower, senior high, and college and above. Personal monthly income (RMB) was categorized into <3,000, 3,000, 5,000, 5,001–10,000, and >10,000. The disease duration of patients with psoriasis was classified as <10, 10–19, 20–29, and ≥30 years.

The PASI was used to evaluate the disease severity of psoriasis in this study. We used the PASI score to evaluate the psoriasis skin disease severity in two main dimensions, including lesion severity and lesion area. Lesion severity is a comprehensive assessment of erythema, desquamation (scale), and infiltration degree of body parts (evaluated respectively), which is divided into 4 points for all three indexes, namely, (I) 0 point, not involved; (II) 1 point, mild; (III) 2 points, moderate; (IV) 3 points, severe; and (V) 4 points, extremely severe. Lesion area score is evaluated in 6 points, namely, 0 point for not involved, 1 point for <10%, 2 point for 10–29%, 3 point for 30–49%, 4 point for 50–69%, 5 point for 70–89%, and 6 point for 90–100%. The weight of body parts is defined as the weight of each body part according to its approximate percentage of the whole body, which is 10% for the head and neck, 20% for the upper limb, 30% for the trunk (e.g., inguinal and axillary), and 40% for lower limb (e.g., hip). In each body part, the sum of erythema (E), desquamation (D), and infiltration (I) severity scores is multiplied by the lesion area score and then multiplied by the weight of the body part to obtain a body part value, and then all four body parts are combined to obtain the total PASI score. The formula for PASI score calculation is the head and neck lesion area ^*^ (E + I + D) ×0.1 + upper limb lesion area ^*^ (E + I + D) ×0.2 + trunk lesion area ^*^ (E + I + D) ×0.3 + lower limb lesion area ^*^ (E + I + D) ×0.4.

The PASI score is the most effective and widely used tool for measuring disease severity in patients with psoriasis, and the PASI score ranges from 0 to 72 with a higher score indicating more severity of psoriasis. In this study, we categorized the PASI score into four categories by its quartile values, namely, <3.0 for Quartile 1 (Q1, P_0_-P_25_), 3.0–7.0 for Quartile 2 (Q2, P_25_-P_50_), 7.1–13.0 for Quartile 3 (Q3, P_50_-P_75_), and over 13.0 for Quartile 4 (Q4, P_75_-P_100_) (22).

### Statistical Analysis

Data analysis in this study was performed using the statistical software SAS 9.4. The quantitative variables with normal distribution were expressed as mean and standard deviation (SD), and a *t*-test was applied to test for significance between different groups. Quantitative variables with skewed distribution were expressed as median and interquartile range (IQR), and a non-parametric rank-sum test was applied to examine the difference between groups. Qualitative variables were presented as frequency counts and proportion (%), and the chi-square test was performed to determine the statistical difference between groups. Logistic regression was applied to calculate the odds ratio (OR) and 95% confidence interval (95% CI) to explore the association between tobacco smoking and disease severity (PASI scores) among patients with psoriasis, with the adjustment of potential confounding factors. In this study, the potential confounding factors were first selected by the univariate analysis with a *p*-value of ≤ 0.05 as the inclusion criteria, and then confirmed by the directed acyclic graphs (DAGs) method. Scatter plot and Spearman correlation (*R*^2^) were used to explore the association between tobacco smoking duration and psoriasis severity among all smokers with psoriasis, and also both in current smokers and former smokers as well for sensitivity analysis. In this study, a *p*-value of <0.05 (two-tailed) was considered statistically significant.

## Results

In this study, we finally included 4,529 patients with psoriasis and classified them into non-smokers (3,134, 69.2%), former smokers (281, 6.2%), and current smokers (1,114, 24.6%). The average age of the 4,529 patients with psoriasis was 38.6 years (SD: 15.5); 64.1% of them were men and 70.4% of them were married. Notably, 33.0% of 4,529 patients with psoriasis had college and above education, nearly half of them were overweight or obese, and ~97% of them had medical insurance. Data in Table 1 indicate that non-smokers were younger than tobacco smokers, the proportion of male and married patients with psoriasis both in former smokers and current smokers was higher than that in non-smokers, respectively, and the differences were all statistically significant (*p* < 0.05). Psoriasis patients without tobacco smoking (non-smokers) had a higher percentage of college and above education, and a lower percentage of overweight and obesity, in comparison with both former smokers and current smokers; the differences were statistically significant (*p* < 0.05).

**Table 1:** The demographic feature of psoriasis patients in China, 2020–2021.

**Variables**	**Total psoriasis patients (*n* = 4,529)**	**Patient with different tobacco smoking status**		
		**Non-smoker** **(*n* = 3,134)**	**Former smoker** **(*n* = 281)**	**Current smoker** **(*n* = 1,114)**
Age (years)^†^, mean (SD)	38.6 (15.5)	36.8 (15.8)	45.9 (15.7)	40.1 (13.5)
Gender^†^, *n* (%)
Male	2,903 (64.1)	1,589 (50.7)	264 (93.9)	1,050 (94.3)
Female	1,626 (35.9)	1,545 (49.3)	17 (6.1)	64 (5.7)
Marital status^†^, *n* (%)
Unmarried	1,339 (29.6)	1,037 (33.1)	49 (17.4)	253 (22.7)
Married	3,190 (70.4)	2,097 (66.9)	232 (82.6)	861 (77.3)
Education^†^, *n* (%)
Junior High and lower	1,553 (34.3)	1,104 (35.2)	94 (33.5)	355 (31.9)
Senior High	1,481 (32.7)	941 (30.0)	100 (35.6)	440 (39.5)
College and above	1,495 (33.0)	1,089 (34.8)	87 (30.9)	319 (28.6)
BMI (kg/m^2^)^†^, median (IQR)	23.7 (21.3–26.5)	23.4 (20.9–26.1)	24.4 (22.2–26.8)	24.5 (22.3–27.4)
BMI (kg/m^2^)^†^, *n* (%)
<18.5 (lower body weight)	283 (6.3)	249 (8.0)	4 (1.4)	30 (2.7)
18.5–23.9 (normal body weight)	2,118 (46.8)	1,530 (48.8)	123 (43.8)	465 (41.7)
24.0–27.9 (overweight)	1,365 (30.1)	866 (27.6)	109 (38.8)	390 (35.0)
≥28 (obesity)	763 (16.9)	489 (15.6)	45 (16.1)	229 (20.6)
Regions^†^, *n* (%)
Northeast of China	541 (11.9)	404 (12.9)	24 (8.5)	113 (10.1)
East of China	838 (18.5)	625 (19.9)	47 (16.7)	166 (14.9)
North of China	1,248 (27.6)	792 (25.3)	77 (27.4)	379 (34.0)
Middle of China	797 (17.6)	554 (17.7)	51 (18.2)	192 (17.2)
South of China	614 (13.6)	425 (13.6)	54 (19.2)	135 (12.1)
Southwest of China	168 (3.7)	135 (4.3)	6 (2.1)	27 (2.4)
Northwest of China	323 (7.1)	199 (6.4)	22 (7.8)	102 (9.2)
Medical insurance, *n* (%)
Yes	4,396 (97.1)	3,031 (96.7)	273 (97.2)	1,092 (98.0)
No	133 (2.9)	103 (3.3)	8 (2.8)	22 (2.0)

† *The differences between groups was statistically significant (P < 0.05)*.

### Tobacco Smoking Condition Among Patients With Psoriasis

Among the 4,529 patients with psoriasis, 1,395 of them were tobacco smokers, and the prevalence of tobacco smoking among patients with psoriasis was 30.8%, with 24.6% for current tobacco smoking and 6.2% for former tobacco smoking. The median value of tobacco smoking years was 13.0 for total smokers (IQR: 8.0–12.0), and approximately one quartile of them had smoking duration over 20 years. The median number of daily consumed cigarettes was 10 with the IQR ranging from 8 to 20, and nearly 70% of them had smoking intensity of <20 cigarettes per day. For former tobacco smokers with psoriasis, the median value of smoking cessation years was 10, with IQR ranging from 1 to 36 years, as shown in Table 2.

**Table 2:** The smoking intensity, smoking duration, tobacco expenses, and smoking cessation attempt among current smokers in rural area of Shanghai, China.

**Variables**	**Total smokers** **(*n* = 1,395)**	**Former smoker** **(*n* = 281)**	**current smokers** **(*n* = 1,114)**	***P***
Years of tobacco smoking (median, IQR)	13.0 (8.0–21.0)	10.0 (5.0–24.0)	13.0 (8.0–20.0)	0.256
Smoking duration (years), *n* (%)				0.053
<10	395 (28.3)	91 (32.4)	304 (27.3)	
10–20	650 (46.6)	113 (40.2)	537 (48.2)	
Over 20	350 (25.1)	77 (27.4)	273 (24.5)	
Daily consumed cigarettes on average^†^ (median, IQR)	10.0 (8.0–20.0)	10.0 (6.0–15.0)	10.0 (8.0–20.0)	0.000
Smoking intensity (cigarettes/day)^†^ *n* (%)				0.000
<20	939 (67.3)	222 (79.0)	717 (64.4)	
Equal or over 20	391 (32.7)	59 (21.0)	397 (35.6)	
Years of smoking cessation (median, IQR)	–	10.0 (1.0–36.0)	–	–
Smoking cessation years, *n* (%)
<10	–	140 (49.8)	–	–
Equal or over 10	–	141 (50.2)	–	–

† *The differences between former smokers and current smokers was statistically significant (P <0.05).*

*IQR, Interquartile Range*.

In comparison with former smokers, current smokers had longer years of tobacco smoking, and the proportion of smoking duration of <10 years was lower in current tobacco smokers, but the differences were not statistically significant (*p* > 0.05). In contrast, current smokers consumed more cigarettes each day on average than former smokers (*p* < 0.05), and the proportion of smoking intensity ≥20 cigarettes/day was statistically higher than that among former smokers (*p* < 0.05), as shown in Table 2.

### Diseases Feature of Patients With Psoriasis

The average PASI score among the 4,529 patients with psoriasis was 9.4 (SD: 8.4), and male patients with psoriasis had a higher PASI score than female patients with psoriasis (*p* < 0.05). For psoriasis lesion severity, the median score was 3.0 (IQR: 0–6) for the head and neck, 4.0 (IQR: 2–6) for the trunk, 4.0 (IQR: 2–6) for the upper limb, and 5.0 (IQR: 3–6) for the lower limb. Meanwhile, male patients had higher psoriasis lesion scores than female patients in all four body parts (*p* < 0.05). Nearly half of the patients with psoriasis in this study had psoriasis aggravation in winter (45.8%), followed by in autumn (20.3%) and spring (15.8%), and male patients had a higher proportion of disease aggravation in winter than female patients (*p* < 0.05). In this study, over 93% of patients had psoriasis vulgaris, and the proportion of psoriasis types was 3.2% for psoriasis erythroderma, 2.6% for psoriasis arthritis, 2.1% for localized pustular psoriasis, and 1.5% for generalized pustular psoriasis. Of note, 16.1% of patients reported psoriasis family history, and the proportion was higher in female patients but without statistical significance, as shown in Table 3.

**Table 3:** The psoriasis severity, family history, seasonality aggravation and skin damage condition among psoriasis patients in China, 2020–2021.

**Variables**	**Total patients** **(*n* = 4,529)**	**Male patients** **(*n* = 2,903)**	**Female patients** **(*n* = 1,626)**	***P***
PASI score^†^, [mean, (SD)]	9.4 (8.4)	10.3 (8.9)	7.8 (7.3)	0.001
PASI score^†^, (median, IQR)	7.0 (3.0–13.1)	7.9 (3.4–14.4)	5.5 (2.4–12.0)	0.001
Psoriasis severity (erythema/infiltration/scales) (median, IQR)
Head total score^†^	3.0 (0.0–6.0)	3.0 (1.0–6.0)	3.0 (0.0–6.0)	0.000
Trunk total score^†^	4.0 (2.0–6.0)	5.0 (2.0–6.0)	4.0 (0.0–6.0)	0.000
Arms total score^†^	4.0 (2.0–6.0)	4.0 (3.0–6.0)	3.0 (2.0–6.0)	0.000
Legs total score^†^	5.0 (3.0–6.0)	6.0 (3.0–7.0)	4.0 (3.0–6.0)	0.000
Seasons of psoriasis aggravation, *n* (%)
Spring	714 (15.8)	443 (15.3)	271 (16.7)	0.213
Summer	361 (8.0)	245 (8.4)	116 (7.1)	0.120
Autumn	919 (20.3)	587 (20.2)	332 (20.4)	0.874
Winter^†^	2,073 (45.8)	1,381 (47.6)	692 (42.6)	0.001
Types of psoriasis, *n* (%)
Psoriasis arthritis	118 (2.6)	82 (2.8)	36 (2.2)	0.216
Psoriasis erythroderma^†^	143 (3.2)	108 (3.7)	35 (2.2)	0.004
Generalized herpetic psoriasis	67 (1.5)	39 (1.3)	28 (1.7)	0.311
Localized herpetic psoriasis^†^	93 (2.1)	48 (1.7)	45 (2.8)	0.011
Psoriasis vulgaris	4,227 (93.3)	2,710 (93.4)	1,517 (93.3)	0.943
Family history of psoriasis patients, *n* (%)				0.533
Yes	727 (16.1)	448 (15.4)	279 (17.2)	
No	3,370 (74.4)	2,186 (75.3)	1,184 (72.8)	
Unknown	432 (9.5)	269 (9.3)	163 (10.0)	
Kins with psoriasis, *n* (%)
Father	224 (4.9)	148 (5.1)	76 (4.7)	0.528
Mother	156 (3.4)	104 (3.6)	52 (3.2)	0.496
Siblings^†^	119 (2.6)	64 (2.2)	55 (3.4)	0.017
Offspring	23 (0.5)	12 (0.4)	11 (0.7)	0.232
Others	228 (5.0)	135 (4.7)	93 (4.5)	0.114

† *The differences between male and female psoriasis patients was statistically significant (P <0.05).*

*IQR, Interquartile Range; SD, Standard Deviation*.

### The Association Between Tobacco Smoking and Psoriasis Severity (PASI Score)

In this study, 4,529 patients with psoriasis were divided into three groups by their tobacco smoking status, and then recombined into condition 1 (non-smoker and former smoker) and condition 2 (non-smoker and current smoker) to explore the association between tobacco smoking and psoriasis severity. In comparison with non-smokers (8.3 ± 7.5), former smokers (13.4 ± 10.3) and current smokers (11.5 ± 9.4) had a higher PASI score, and the differences were statistically significant. The proportion of PASI score over 7 was 44.2% in non-smokers, which was obviously lower than that in former smokers (66.9%), and in current smokers as well (58.1%). For psoriasis lesion severity score, former smokers had higher scores than non-smokers in all four body parts, and the differences were all statistically significant, as shown in Table 4.

**Table 4:** The association between tobacco smoking and psoriasis severity (PASI score) among psoriasis patients in China, 2020–2021.

**Variables**	**Condition 1**		**Condition 2**	
	**Non-smoker** **(*n* = 3,134)**	**Former smoker** **(*n* = 281)**	**Non-smoker** **(*n* = 3,134)**	**Current smoker** **(*n* = 1,114)**
PASI score^†^^‡^ [mean (SD)]	8.3 (7.5)	13.4 (10.3)	8.3 (7.5)	11.5 (9.4)
PASI score^†^^‡^ (median, IQR)	6.0 (2.7–12.9)	11.0 (5.4–19.2)	6.0 (2.7–12.9)	9.0 (4.2–16.4)
PASI score classification in quartile^†^^‡^, *n* (%)
<3.0 (Quartile 1, Q1)	927 (29.6)	40 (14.2)	927 (29.6)	180 (16.2)
3.0–7.0 (Quartile 2, Q2)	823 (26.3)	53 (18.9)	823 (26.3)	287 (25.8)
7.1–13.0 (Quartile 3, Q3)	742 (23.7)	71 (25.3)	742 (23.7)	262 (23.5)
Over 13.0 (Quartile 4, Q4)	642 (20.5)	117 (41.6)	642 (20.5)	385 (34.6)
Psoriasis severity score (median, IQR)
Head total score^†^^‡^	3.0 (0.0–6.0)	4.0 (1.0–6.0)	3.0 (0.0–6.0)	3.0 (1.0–6.0)
Trunk total score^†^^‡^	4.0 (1.0–6.0)	5.0 (3.0–7.0)	4.0 (1.0–6.0)	4.0 (2.0–6.0)
Arms total score^†^^‡^	4.0 (2.0–6.0)	4.0 (3.0–6.0)	4.0 (2.0–6.0)	5.0 (3.0–6.0)
Legs total score^†^^‡^	5.0 (3.0–6.0)	6.0 (3.0–7.0)	5.0 (3.0–6.0)	6.0 (3.0–7.0)

† *The difference between former smokers and non-smokers was statistically significant (P <0.05).*

‡ *The difference between current smokers and non-smokers was statistically significant (P <0.05).*

*Quartile, Q1 (P_0_-P_25_), Q2 (P_25_-P_50_), Q3 (P_50_-P_75_), Q4 (P_75_-P_100_); SD, standard deviation; IQR, Interquartile Range*.

**Figure 2:**
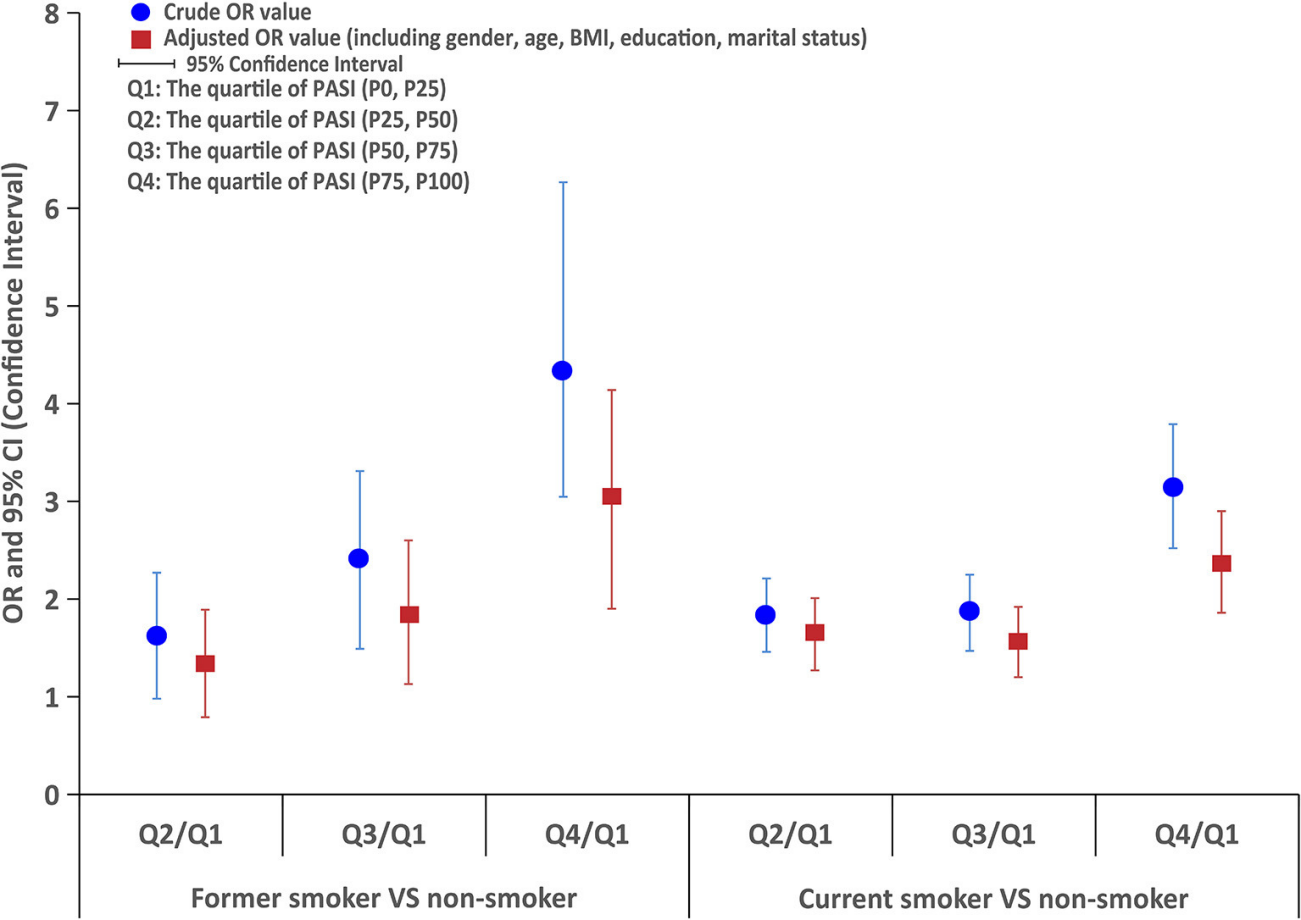
The logistic regression analysis of PASI score between former smokers or current smokers and non-smokers.

The logistic regression analysis indicated that former smokers had higher PASI scores than non-smokers, and the OR was 1.5 (95% CI: 1.0–2.3) for PASI score (3.0–7.0) compared with PASI score <3.0, 2.2 (95% CI: 1.5–3.3) for PASI score (7.1–13.0) compared with PASI score <3.0, and 4.2 (95% CI: 2.9–6.1) for PASI score > 13 compared with PASI score <3.0, even with the adjustment of potential confounding factors (ORs were 1.2, 1.7, and 2.8, respectively). Similarly, current smokers also had higher PASI scores than non-smokers, and logistic regression showed that the OR was 1.8 (95% CI: 1.5–2.2) for PASI score (3.0–7.0) compared with PASI score <3.0, 1.9 (95% CI: 1.5–2.3) for PASI score (7.1–13.0) compared with PASI score <3.0, and 3.1 (95% CI: 2.5–3.8) for PASI score >13 compared with PASI score <3.0, even with the adjustment of potential confounding factors (ORs were 1.6, 1.5, and 2.3, respectively), as shown in Figure 2.

**Figure 3:**
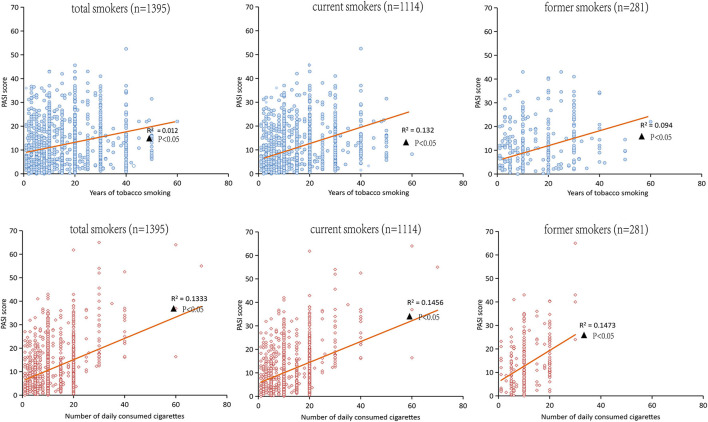
Spearman correlation analysis for the PASI score and the years of tobacco or the numbers of daily consumed cigarettes in smokers.

Among the 1,395 patients with psoriasis who were tobacco smokers, the Spearman correlation analysis in Figure 3 indicated that the PASI score was positively correlated with tobacco smoking years among smokers, and the findings were consistent both in current smokers and former smokers (*p* < 0.05). Meanwhile, the Spearman correlation analysis also demonstrated that the PASI score was positively correlated with the number of daily consumed cigarettes both in current smokers and former smokers (*p* < 0.01), as shown in Figure 3.

## Discussion

This is the first large population size study in China that enrolled 4,529 patients with psoriasis to explore the association between tobacco smoking and disease severity of psoriasis. This study indicated that the prevalence of tobacco smoking among patients with psoriasis was 30.8%, with 24.6% for current tobacco smoking and 6.2% for former tobacco smoking, which was in line with the findings in previous studies (23, 24). In this study, the average PASI score among the patients with psoriasis was 9.4 (SD: 8.4), and male patients had a higher PASI score than female patients. Logistic regression analysis indicated that both the former smokers and current smokers had at least 1.2 times higher PASI scores than non-smokers, and PASI score was positively correlated with tobacco smoking years as well as number of daily consumed cigarettes. Therefore, we concluded that tobacco smoking was positively associated with the disease severity of psoriasis, and both the tobacco smoking duration and smoking intensity were positively correlated with the psoriasis severity among patients with tobacco smoking. These findings have important implications concerning the management and treatment of psoriasis patients, meanwhile, providing direct evidence to support advocating smoking cessation among patients with psoriasis. Therefore, doctors should promote tobacco control measures, such as pharmacotherapy, smoking cessation advice, and behavioral support, and provide assistance for smoking cessation among patients with psoriasis. Moreover, the effect of doctors' advice and assistance for tobacco smoking cessation should be evaluated among patients with psoriasis in the future, both for the direct effect of decreased smoking prevalence, and the recurrence and severity of psoriasis.

Growing evidence demonstrates the association between tobacco smoking and the onset and development of psoriasis. Therefore, it is important to understand the tobacco smoking status in patients with psoriasis. Previous evidence has indicated that the prevalence of tobacco smoking varies substantially in different studies. Kridin et al. (25) implemented an investigation that enrolled 121,801 patients with psoriasis, of whom 38.9% were tobacco smokers. A cross-sectional study in Korea with 1,260 patients with psoriasis showed a 51.2% of tobacco smoking prevalence (26); meanwhile, a meta-analysis with pooled data indicated a 30.04% of tobacco smoking prevalence among patients with psoriasis (20). In this study, the prevalence of tobacco smoking was 30.8%, which was consistent with the finding (33.4% of tobacco smoking prevalence) of a recent multicenter study in China (27). The low tobacco smoking prevalence in the Chinese psoriasis population might partially attribute to a lower prevalence of tobacco smoking among female patients with psoriasis. In this study, the prevalence of tobacco smoking was 5.0% among female patients, which was extremely lower than that of Fortes' survey (50% of female patients with psoriasis were smokers) (28). Meanwhile, a growing number of population in China have noticed the physical harmfulness of tobacco use in recent years and stopped smoking successfully, which might also contribute to the lower prevalence of tobacco smoking in this study (29). In contrast, we should notice the high prevalence of tobacco smoking in men, especially with senior high education; we suggest that tobacco control measures should be focused on this specific population in the future.

In clinical practice, psoriasis severity is often categorized as mild, moderate, and severe. Although there were numerous and reliable tools to assess the psoriasis severity, PASI was the primary tool for disease severity evaluation to reflect the treatment response in patients with psoriasis by a dermatologist. Meanwhile, we also emphasized that evaluators should consider the type and location of lesions among patients with psoriasis in order to assess the severity of skin lesions more accurately. In this study, the average PASI score among the patients with psoriasis was 9.4 that indicated mild to moderate psoriasis severity, and male patients with psoriasis had a higher PASI score than female patients; this might be partially due to the higher tobacco smoking prevalence among male patients. In this study, we also noticed that over 93% of patients suffered from psoriasis vulgaris, and psoriasis lesion severity scores were higher for lower limbs among patients, which was in line with the findings of the nationwide survey in China during 2009 and 2010 (23). In contrast, investigation in Western countries indicated that the most frequently seriously affected areas are knees, scalp, body trunk, and hands (30, 31). These inconsistent findings indicated the multifacet variation of psoriasis phenotype, so a better understanding of the distribution and impact of different body areas for psoriasis is crucial for disease management.

Tobacco smoking is one of the well-established environmental risk factors for psoriasis, but its association with psoriasis severity is still limited. A meta-analysis shows that both current smokers [1.63 (95% CI: 1.48–1.80)] and former smokers [1.36 (95% CI: 1.13–1.64)] have a higher risk of developing psoriasis than nonsmokers (32). Other studies indicated that 15–20% of psoriasis onset could attribute to the former or current tobacco smoking, suggesting that tobacco smoking increased the risk of psoriasis both in women and men (33). This study showed that tobacco smoking impacts the severity of psoriasis in a dose-dependent fashion, and tobacco smoking intensity and smoking duration were positively associated with the severity of psoriasis, which were in line with the findings in the previous studies (34). Meanwhile, tobacco smoking might also directly impact the treatment response and lower the adherence to therapy among patients with psoriasis, leading to the aggravation of psoriasis severity in patients. In comparison with non-smokers, former smokers were less likely to achieve disease improvement with biologic agent treatment. Interestingly, in a series of 110 psoriasis patients treated with anti-TNF therapy, the majority of non-responders were smokers, especially those with overweight/obesity or higher baseline PASI scores (35). In this study, both the former smokers and current smokers had higher PASI scores, which predicted the lower response rate of treatment in these patients with tobacco smoking, so implementing tobacco control measures among patients with psoriasis is crucial for the improvement of treatment response in the future.

Tobacco smoking as a complex risk factor might influence the onset and development of psoriasis in many ways, but the underlying mechanism is still not clear. The pathophysiological mechanisms for the direct association between psoriasis and tobacco smoking include oxidative stress, free radical damage, increasing vascular endothelial dysfunction, immune cell activation, genetic mechanisms and interaction with key signaling pathways in psoriasis [nuclear factor kappa B (NF-κB), Janus kinase/signal transducers and activators of transcription (JAK-STAT)], and mitogen-activated protein kinase (MAPK) (36–38). The consequences of oxidative stress and free radical damage had been shown to transform the morphology and function of psoriatic polymorphonuclear cells, which comprised the dominant inflammatory infiltrate in psoriasis (28). In addition, it was also suggested that tobacco smoke could increase vascular endothelial dysfunction, which is an important factor for angiogenesis of psoriasis (39). Nicotine in tobacco smoke was the major alkaloid of cigarettes that activated the interleukin-1β (IL-1β), IL-2, IL-12, and tumor necrosis factor (TNF), which played a central role in psoriasis pathogenesis (40, 41). Presumably, nicotine might facilitate keratinocyte adhesion and upward migration in the epidermis and eventually impacted keratinocyte proliferation and skin inflammation (42). Nicotine also stimulated innate immune cells, including keratinocytes, macrophages, and dendritic cells (32). These cells release cytokines that trigger T-lymphocytes and perpetuate the cycle of chronic inflammation, leading to the onset and development of psoriasis.

A key strength of this study is the large population size of the patient with psoriasis. We sampled 4,529 patients from 200 hospitals, which accounted for ~2% of all 12,436 tertiary hospitals and secondary hospitals in China, so the findings could be generalized to patients with psoriasis in China. Meanwhile, the data collection was conducted by an electronic software that was convenient to the paperless data input, the full-time audio record benefit the subsequent inspection, and the clinical data of psoriasis patients were extracted from the health information system (HIS) directly without recall bias, which ensures the high quality of data is another strength of this study.

There are some limitations in this study. First, tobacco smoking status among patients is self-reported which might lead to recall bias, and the missing information of second-hand smoke exposure might underestimate the influence of tobacco smoking on psoriasis severity to some degree. Second, indicators for life quality assessment are not included in this study, so we missed the chance to evaluate the influence of psoriasis severity for life quality of patients with and without tobacco smoking; the incorporation of Physician's Global Assessment (PGA), Dermatology Life Quality Index (DLQI), Professional Quality of Life Scale (PRQoL) and Visual Analog Scale (VAS) should be considered in future study. Third, the nature of cross-sectional study design only allows the calculation of tobacco smoking prevalence and estimation of the correlation between tobacco smoking and psoriasis severity, but not the causal relationship. All of these aforementioned limitations would restrict the interpretations of clinical findings to some degree. Therefore, a better designed cohort or case–control study to explore the potential mechanism of smoking behavior on diseases severity among patients with psoriasis may be particularly useful for the development of future research directions and intervention strategies.

## Conclusion

The prevalence of tobacco smoking among patients with psoriasis was high, especially among male patients and those with senior high education. Tobacco smoking was positively associated with psoriasis severity; moreover, both smoking intensity and smoking duration were positively correlated with the severity of psoriasis in a dose-dependent fashion.

## Data Availability Statement

The raw data supporting the conclusions of this article will be made available by the authors, without undue reservation.

## Ethics Statement

The studies involving human participants were reviewed and approved by Institutional Review Board of Peking University First Hospital and Shanghai Skin Diseases Hospital. The patients/participants provided their written informed consent to participate in this study.

## Author Contributions

RW and BL proposed and designed the study. LW, SC, ZZ, LK, and RZ collected the data. NY and RW performed the statistical analyses. WL and SC drafted the manuscript. ZZ, YS, and BL supervised the study. RW revised the manuscript. All authors contributed to the article and approved the submitted version.

## Funding

This study was supported by the National Key Research and Development Program of China (No. 2018YFC1705301), and the NSFC of China (Nos. 81973860, 81874470, 82074427, and 81904214), and Intelligence Funds of Shanghai Skin Disease Hospital (2021KYQD01), the Shanghai Talent Development Fund (2021073), the Shanghai Shenkang Hospital Development Center Management Research Program (2020SKMR-32), the Shanghai Sailing Program (No. 21YF1448100), the Xinglin Youth Scholar of Shanghai University of Traditional Chinese Medicine (No. RY411.33.10), and the Shanghai Development Office of TCM (No. ZY(2018-2020)-FWTX-1008 and ZY(2018-2020)-FWTX-4010), the Shanghai Municipal Key Clinical Specialty (No. shslczdzk05001), and the Shanghai Pujiang Talent Program (No. 2020PJD067). These funders had no role in study design, data collection and analysis, decision for publication, or preparation of the manuscript.

## Conflict of Interest

The authors declare that the research was conducted in the absence of any commercial or financial relationships that could be construed as a potential conflict of interest.

## Publisher's Note

All claims expressed in this article are solely those of the authors and do not necessarily represent those of their affiliated organizations, or those of the publisher, the editors and the reviewers. Any product that may be evaluated in this article, or claim that may be made by its manufacturer, is not guaranteed or endorsed by the publisher.

## Abbreviations

HIS: Health Information System
PASI: Psoriasis Area and Severity Index
OR: Odds Ratios
CI: Confidence Interval
GATS: Global Adult Survey
NCRCSID: National Clinical Research Center for Skin and Immune Diseases
BMI: body mass index
SD: Standard Deviation
IQR: Interquartile Range
DAGs: Directed Acyclic Graphs
NF-κB: nuclear factor kappa B
JAK-STAT: Janus kinase/Signal transducers and activators of transcription
MAPK: mitogen-activated protein kinase
IL-1β: interleukin-1β
IL-2: interleukin-2
IL-12: interleukin-12
TNF: tumor necrosis factor
PGA: Physician's Global Assessment
DLQI: Dermatology Life Quality Index
PRQoL: Professional Quality of Life Scale
VAS: Visual Analog Scale.

## References

[1] Zeng J Luo S Huang Y Lu Q. Critical role of environmental factors in the pathogenesis of psoriasis. J Dermatol. (2017) 44:863–72. 10.1111/1346-8138.13806 28349593

[2] Greb JE Goldminz AM Elder JT Lebwohl MG Gladman DD Wu JJ. Psoriasis. Nat Rev Dis Primers. (2016) 2:16082. 10.1038/nrdp.2016.82 27883001

[3] Michalek IM Loring B John SM. A systematic review of worldwide epidemiology of psoriasis. J Eur Acad Dermatol Venereol. (2017) 31:205–12. 10.1111/jdv.13854 27573025

[4] Armstrong AW Read C. Pathophysiology, clinical presentation, and treatment of psoriasis: a review. JAMA. (2020) 323:1945. 10.1001/jama.2020.4006 32427307

[5] Shao CG Zhang GW Wang GC. Distribution of psoriasis in China: a nationwide screening. Proc Chin Acad Med Sci Peking Union Med Coll. (1987) 2:59–65. 3423009

[6] Ding X Wang T Shen Y Wang X Zhou C Tian S. Prevalence of psoriasis in China: a population-based study in six cities. Eur J Dermatol. (2012) 22:663–7. 10.1684/ejd.2012.1802 22910173

[7] Griffiths CE Barker JN. Pathogenesis and clinical features of psoriasis. Lancet. (2007) 370:263–271. 10.1016/S0140-6736(07)61128-3 17658397

[8] Korman NJ. Management of psoriasis as a systemic disease: what is the evidence? Br J Dermatol. (2020) 182:840–8. 10.1111/bjd.18245 31225638 PMC7187293

[9] Michelsen B Uhlig T Sexton J Kristianslund EK Wierød A Bakland G. Healthrelated quality of life in patients with psoriatic and rheumatoid arthritis: data from the prospective multicenter NOR-DMARD study compared with Norwegian general population controls. Ann Rheum Dis. 77:1290–4. 10.1136/annrheumdis-2018-213286 29875096

[10] Mattei PL Corey KC Kimball AB. Psoriasis Area Severity Index (PASI) and the Dermatology Life Quality Index (DLQI): the correlation between disease severity and psychological burden in patients treated with biological therapies. J Eur Acad Dermatol Venereol. (2014) 28:333–7. 10.1111/jdv.12106 23425140

[11] Pilon D Teeple A Zhdanava M Ladouceur M Ching Cheung H Muser E. The economic burden of psoriasis with high comorbidity among privately insured patients in the United States. J Med Econ. (2019) 22:196–203. 10.1080/13696998.2018.1557201 30523738

[12] Kobayashi K Kamekura R Kato J Kamiya S Kamiya T Takano K. Cigarette smoke underlies the pathogenesis of palmoplantar pustulosis via an IL-17A–induced production of IL-36γ in tonsillar epithelial cells. J Invest Dermatol. (2021) 141:1533–41.e4. 10.1016/j.jid.2020.09.028 33188781

[13] Pezzolo E Naldi L. The relationship between smoking, psoriasis and psoriatic arthritis. Expert Rev Clin Immunol. (2019) 15:41–8. 10.1080/1744666X.2019.1543591 30380949

[14] Bertesi M Fantini S Alecci C Lotti R Martello A Parenti S. Promoter methylation leads to decreased ZFP36 expression and deregulated NLRP3 inflammasome activation in psoriatic fibroblasts. Front Med. (2021) 7:579383. 10.3389/fmed.2020.579383 33585499 PMC7874095

[15] Lipa K Zajac N Owczarek W Ciechanowicz P Szymańska E Walecka I. Does smoking affect your skin? Pdia. (2021) 38:371–6. 10.5114/ada.2021.103000 34377115 PMC8330869

[16] Salihbegovic E Kurtalic N Omerk E. Smoking cigarettes and consuming alcohol in patients with psoriasis. Mater Sociomed. (2021) 33:30. 10.5455/msm.2021.33.30-33 34012347 PMC8116091

[17] Richard E. ABC of smoking cessation: the problem of tobacco smoking. BMJ. (2004) 328: 217. 10.1136/bmj.328.7433.217

[18] Liu S Zhang M Yang L Li Y Wang L Huang Z. Prevalence and patterns of tobacco smoking among Chinese adult men and women: findings of the 2010 national smoking survey. J Epidemiol Community Health. (2017) 71:154–61. 10.1136/jech-2016-207805 27660401 PMC5284482

[19] Morton J Song Y Fouad H Awa FE Abou El Naga R Zhao L. Cross-country comparison of waterpipe use: nationally representative data from 13 low and middle-income countries from the Global Adult Tobacco Survey (GATS). Tob Control. (2014) 23:419–27. 10.1136/tobaccocontrol-2012-050841 23760609 PMC4145417

[20] Groot J Nybo Andersen AM Blegvad C Pinot de Moira A Skov L. Prenatal, infantile, and childhood tobacco exposure and risk of pediatric psoriasis in the Danish National Birth Cohort offspring. J Am Acad Dermatol. (2020) 83:1625–32. 10.1016/j.jaad.2019.09.038 31973955

[21] Dai YX Wang SC Chou YJ Chang YT Chen TJ Li P. Smoking, but not alcohol, is associated with risk of psoriasis in a Taiwanese population-based cohort study. J Am Acad Dermatol. (2019) 80:727–34. 10.1016/j.jaad.2018.11.015 30528570

[22] Lee EJ Han KD Han JH Lee JH. Smoking and risk of psoriasis: a nationwide cohort study. J Am Acad Dermatol. (2017) 77:573–5. 10.1016/j.jaad.2017.04.015 28807112

[23] Mrowietz U Mrowietz U Menter A Griffiths CEM Bagel J Strober B. Effect of baseline disease severity on achievement of treatment target with apremilast: results from a pooled analysis. J Eur Acad Dermatol Venereol. (2021) 35:2409–14. 10.1111/jdv.17520 34255891

[24] Richer V Roubille C Fleming P Starnino T McCourt C McFarlane A. Psoriasis and smoking: a systematic literature review and meta-analysis with qualitative analysis of effect of smoking on psoriasis severity. J Cutan Med Surg. (2016) 20:221–7. 10.1177/1203475415616073 26553732

[25] Kridin K Linder D Shalom G Piaserico S Babaev M Freud T. Psoriasis and dementia: a cross-sectional study of 121,801 patients. Acta Derm Venereol. (2020) 100:adv00250. 10.2340/00015555-3595 32725254 PMC9207629

[26] Youn SW Lee JH Yu DY Kim Y Kim BS Seo SJ. The relationship between clinical characteristics including presence of exposed lesions and health-related quality of life (HRQoL) in patients with psoriasis: analysis from the nationwide epidemiologic study for psoriasis in Korea (EPI-PSODE study). J Eur Acad Dermatol Venereol. (2018) 32:1499–506. 10.1111/jdv.14865 29430733

[27] Chen K Wang G Jin H Xu J Zhu X Zheng M. Clinic characteristics of psoriasis in China: a nationwide survey in over 12000 patients. Oncotarget. (2017) 8:46381–9. 10.18632/oncotarget.18453 28637026 PMC5542274

[28] Fortes C Mastroeni S Leffondré K Sampogna F Melchi F Mazzotti E. Relationship between smoking and the clinical severity of psoriasis. Arch Dermatol. (2005) 141:5. 10.1001/archderm.141.12.1580 16365261

[29] Xiao D Kotler M Kang J Wang C. A multicenter, randomized, double-blind, parallel, placebo-controlled clinical study to evaluate the efficacy and safety of a nicotine mint lozenge (2 and 4 mg) in smoking cessation. J Addict Med. (2020) 14:69–77. 10.1097/ADM.0000000000000547 31658113 PMC7012347

[30] Augustin M Sommer R Kirsten N Danckworth A Radtke MA Reich K. Topology of psoriasis in routine care: results from high-resolution analysis of 2009 patients. Br J Dermatol. (2019) 181:358–65. 10.1111/bjd.17403 30430557

[31] Hawro M Maurer M Weller K Maleszka R Zalewska-Janowska A Kaszuba A. Lesions on the back of hands and female gender predispose to stigmatization in patients with psoriasis. J Am Acad Dermatol. (2017) 76:648–54.e2. 10.1016/j.jaad.2016.10.040 28069297

[32] Zhou H Wu R Kong Y Zhao M Su Y. Impact of smoking on psoriasis risk and treatment efficacy: a meta-analysis. J Int Med Res. (2020) 48:030006052096402. 10.1177/0300060520964024 33121308 PMC7780610

[33] Armstrong AW Harskamp CT Dhillon JS Armstrong EJ. Psoriasis and smoking: a systematic review and meta-analysis. Br J Dermatol. (2014) 170:304–14. 10.1111/bjd.12670 24117435

[34] Li W Han J Choi HK Qureshi AA. Smoking and risk of incident psoriasis among women and men in the united states: a combined analysis. Am J Epidemiol. (2012) 175:402–13. 10.1093/aje/kwr325 22247049 PMC3329197

[35] Di Lernia V Ricci C Lallas A Ficarelli E. Clinical predictors of non-response to any tumor necrosis factor (TNF) blockers: a retrospective study. J Dermatol Treat. (2014) 25:73–4. 10.3109/09546634.2013.800184 23621374

[36] Naldi L. Psoriasis and smoking: links and risks. Psoriasis. (2016) 6:65–71. 10.2147/PTT.S85189 29387595 PMC5683129

[37] Yanagita M Kobayashi R Kojima Y Mori K Murakami S. Nicotine modulates the immunological function of dendritic cells through peroxisome proliferator-activated receptor-γ upregulation. Cell Immunol. (2012) 274:26–33. 10.1016/j.cellimm.2012.02.007 22425227

[38] Armstrong AW Armstrong EJ Fuller EN Sockolov ME Voyles SV. Smoking and pathogenesis of psoriasis: a review of oxidative, inflammatory and genetic mechanisms: smoking and pathogenesis of psoriasis. Br J Dermatol. (2011) 165:1162–8. 10.1111/j.1365-2133.2011.10526.x 21777217

[39] Csordas A Bernhard D. The biology behind the atherothrombotic effects of cigarette smoke. Nat Rev Cardiol. (2013) 10:219–30. 10.1038/nrcardio.2013.8 23380975

[40] Gazel U Ayan G Solmaz D Akar S Aydin SZ. The impact of smoking on prevalence of psoriasis and psoriatic arthritis. Rheumatology. (2020) 59:2695–710. 10.1093/rheumatology/keaa179 32500136

[41] Nestle FO Kaplan DH Barker J. Psoriasis. N Engl J Med. (2009) 361:496–509. 10.1056/NEJMra0804595 19641206

[42] Fowles J. Application of toxicological risk assessment principles to the chemical constituents of cigarette smoke. Tobacco Control. (2003) 12:424–30. 10.1136/tc.12.4.424 14660781 PMC1747794

